# Emerging Roles of Surfactants in Engineering Two-Dimensional Nanomaterials for Advanced Applications

**DOI:** 10.3390/nano16211426

**Published:** 2026-11-05

**Authors:** Rojoba Yasmin, Jabia Mostofa Chowdhury, Sultana Razia Akhter, Sk. Md. Golam Mostafa

**Affiliations:** 1 Resch School of Engineering, University of Wisconsin–Green Bay, Green Bay, WI 54311, USA; 2 Department of Electrical Engineering, Texas A&M University–Texarkana, Texarkana, TX 75503, USA; jchowdhury@tamut.edu; 3 Independent Researcher, Phoenix, AZ 85142, USA; razia.sub.26@gmail.com; 4 Department of Electrical and Electronic Engineering, International Islamic University Chittagong, Chattogram 4318, Bangladesh; mostafa93@iiuc.ac.bd

**Keywords:** two-dimensional (2D) nanomaterial, surfactant, drug delivery, morphology, synthesis, environmental remediation

## Abstract

Two-dimensional (2D) nanomaterials offer promising properties for energy storage, drug delivery, and environmental remediation, but their performance can be limited by aggregation, restacking, and challenges in controlling morphology and surface properties. Surfactants provide versatile tools for addressing these limitations through surface adsorption, template-directed growth, exfoliation, and dispersion stabilization. This review critically examines how surfactant-assisted strategies influence the synthesis, morphology, interfacial properties, and stability of 2D nanomaterials and how these changes relate to application performance. Representative studies are considered across drug delivery, energy storage, and environmental remediation, with attention to reported outcomes such as pollutant adsorption, oil–water separation, electrical conductivity, and photocatalytic activity. Where available, quantitative results are compared across representative studies, highlighting reported performance improvements and the limitations of cross-study comparisons. The review also distinguishes the direct effects of surfactant–material interactions from performance changes associated with morphology, surface chemistry, defects, and interlayer structure. Practical surfactant selection requires balancing dispersion and structural control against potential active-site blockage, impaired charge or ion transport, purification demands, environmental concerns, and production costs. Residual surfactants, inconsistent processing, limited reproducibility, and scalability remain important challenges. By connecting processing mechanisms with application outcomes and practical limitations, this review identifies priorities for more systematic surfactant selection, improved process control, and environmentally responsible, reproducible production of 2D nanomaterials.

## 1. Introduction

Two-dimensional nanomaterials, which are only a single atom or a few atoms thick, are revolutionizing science and technology by breaking the physical limits of traditional bulk materials. They have attracted significant interest in a variety of research fields because of their unique structural, chemical, electrical, and optical characteristics. Since monolayer graphene was successfully separated from graphite in 2004 [1], the special properties of 2D materials have drawn considerable attention. Examples of such materials include graphene, transition metal dichalcogenides (TMDs), MXenes, hexagonal boron nitride (h-BN), black phosphorus (BP), and 2D metal–organic frameworks (MOFs) [1,2,3]. These materials have been extensively studied for applications including energy storage, sensing, catalysis, drug delivery, and environmental remediation. Because of their extremely small thickness, large surface area, high aspect ratio, and tunable surface chemistry, they are suitable for a wide range of applications.

Although 2D materials have contributed significantly to many applications, they also face several challenges. For example, their large surface area and high surface energy can cause the sheets to naturally restack. In addition, it can be challenging to control the thickness, size, morphology, crystal growth, and dispersion during synthesis. Therefore, different approaches have been developed to overcome these limitations and improve the stability and performance of 2D nanomaterials. Studies show that adding surfactants during the synthesis or modification of 2D materials can help solve some of these problems and enhance the final product’s performance [4,5,6,7]. For example, surfactants can improve the dispersion of 2D nanomaterials by modifying their surface properties and making hydrophobic materials more compatible with the surrounding solvent. Sodium dodecyl sulfate (SDS), Cetyltrimethylammonium bromide (CTAB), and sodium cholate (SC) have been used to disperse transition metal chalcogenides in water [7].

Surfactants are amphiphilic molecules that contain both hydrophilic and hydrophobic parts, allowing them to interact with both the surrounding solvent and the surface of nanomaterials [8]. Depending on their chemical structure and concentration, surfactants can interact with the surface of 2D materials and influence their growth, morphology, thickness, dispersion, and surface properties. During synthesis, surfactants can interact with specific crystal surfaces or metal ions and affect the growth direction of the material. They can also prevent the aggregation and restacking of nanosheets by providing interactions between the material surface and the surrounding medium. Although many studies have reported the benefits of surfactants in 2D nanomaterials, their roles and effects vary with the surfactant type, material, and processing conditions. Furthermore, improvements in material performance may arise not only from direct surfactant–material interactions but also from associated changes in morphology, surface chemistry, defects, interlayer spacing, or interfacial structure. These factors make it important to distinguish the functions of surfactants during synthesis, exfoliation, and stabilization from their subsequent effects on material properties and application performance.

This review focuses on surfactants as engineering tools for controlling the synthesis, morphology, dispersion, and surface properties of 2D nanomaterials. It connects surfactant-assisted processing strategies with their proposed mechanisms, reported performance outcomes, and practical limitations. Applications in drug delivery, energy storage, and environmental remediation are examined alongside the roles of residual surfactants, purification, toxicity, reproducibility, and scalability. Quantitative comparisons of representative studies are also used, where data are available, to highlight performance differences and the limitations of comparing results obtained under different experimental conditions. By integrating these aspects, this review aims to provide a critical perspective on surfactant selection and processing strategies for developing 2D nanomaterials with controlled properties and improved application performance.

This article presents a narrative review of surfactant-assisted engineering of two-dimensional (2D) nanomaterials. Relevant literature was identified through topic-based searches of Google Scholar, Google, and PubMed. The searches focused on combinations of keywords related to nanomaterials, 2D materials, surfactants, and specific applications, including “2D materials AND surfactants AND environmental remediation,” “2D materials AND surfactants AND energy storage,” and corresponding combinations for drug delivery, exfoliation, dispersion, stabilization, and morphology control. The review prioritizes recent literature reflecting developments in this evolving field while including earlier foundational studies where relevant. The last literature search was conducted in August 2026. Original research articles and relevant review papers were considered if they addressed surfactant-assisted synthesis, exfoliation, stabilization, surface modification, mechanisms, or application performance. Publications were selected based on their relevance to the review scope and their contribution to understanding surfactant–material interactions and structure–property relationships. Studies outside the scope of surfactant-assisted engineering of 2D nanomaterials, duplicate records, and publications lacking sufficient relevant information were excluded. As a narrative review, the literature was critically synthesized to identify key developments, compare reported findings, and distinguish experimentally demonstrated surfactant effects from proposed mechanisms and effects associated with changes in material structure or composition.

Previous reviews have addressed complementary aspects of two-dimensional (2D) materials research. Dong et al. [9] reviewed interface-assisted synthesis strategies for 2D materials, including approaches that use interfaces to guide material formation. Chavalekvirat et al. [10] focused on liquid-phase exfoliation, examining how processing parameters, including surfactant selection, influence nanosheet yield, structural and chemical properties, and electrochemical applications. Together, these reviews provide important perspectives on 2D-material synthesis and processing, but their primary emphases differ from the cross-application analysis presented here. The present review connects surfactant roles across morphology control, interfacial interactions, liquid-phase exfoliation, dispersion stabilization, and structural organization with reported performance in energy storage, environmental remediation, and biomedical applications. It also critically examines the trade-offs associated with surfactant retention and removal, distinguishing experimentally demonstrated surfactant contributions from effects that may arise from material morphology, defects, hybridization, or processing conditions.

**Figure 1:**
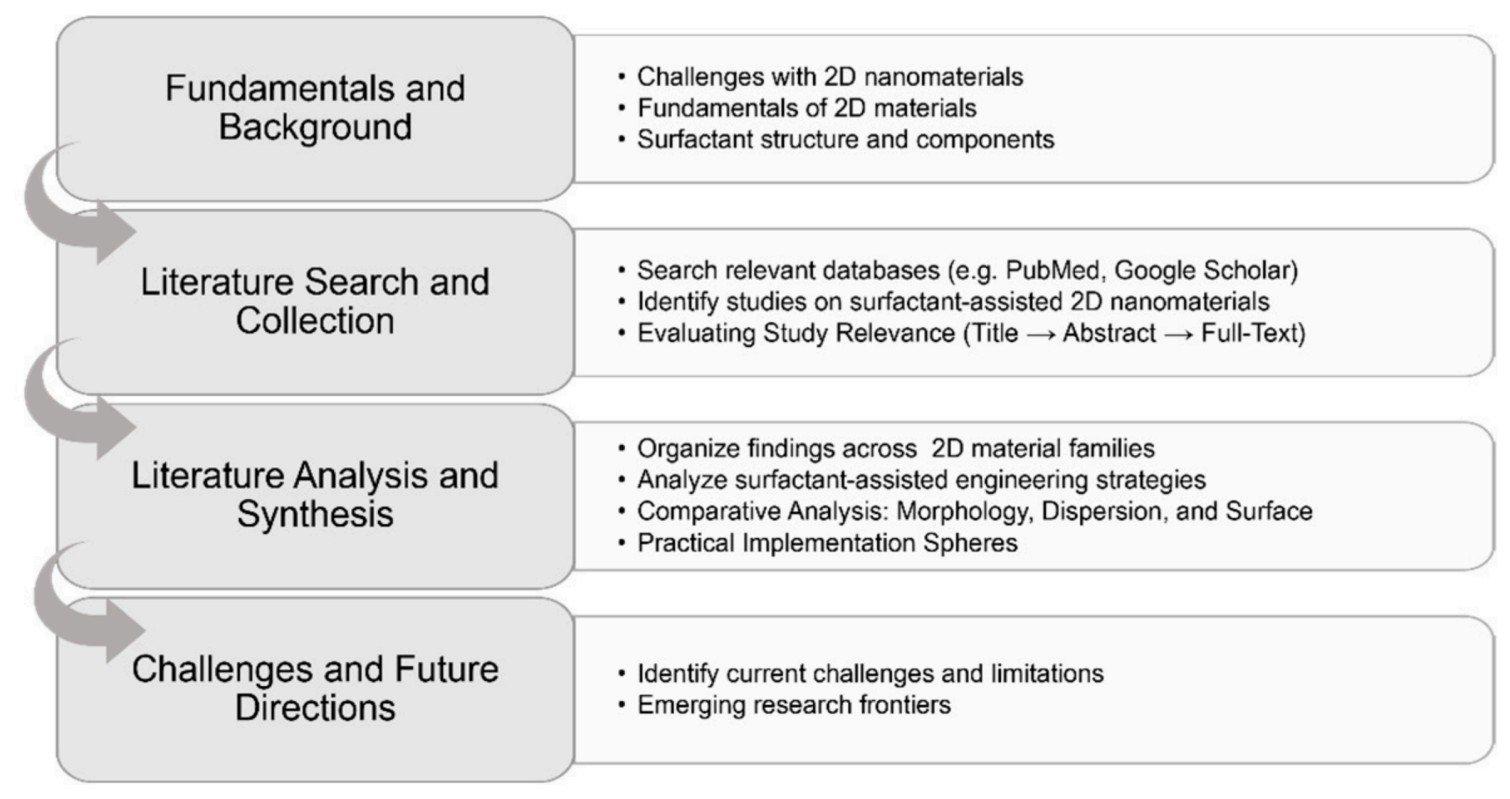
Structural workflow and methodology framework of the review.

To ensure a comprehensive synthesis of the literature, the structural organization and methodology of this review paper are organized according to the roadmap presented in Figure 1. Section 2 introduces the main types, properties, and synthesis approaches of 2D nanomaterials, followed by the fundamental characteristics of surfactants in Section 3. Section 4 discusses how surfactants are used during the synthesis and engineering of 2D nanomaterials, including their roles in morphology control, exfoliation, and dispersion. The major applications of these materials, including energy storage, drug delivery, and environmental remediation, are discussed in Section 5. Section 6 presents the current challenges and future prospects of surfactant-assisted 2D nanomaterials, followed by the conclusions in Section 7.

## 2. Fundamentals of Two-Dimensional Nanomaterials

In this advancing era of nanotechnology, nanoscale materials have become critically important because of their multidisciplinary applications in many research fields, including biomedicine, nanomanufacturing, electronics, drug delivery, energy storage, and more. In particular, 2D materials have gained the most attention due to their unique properties. 2D materials have a nanoscale size in only one dimension. They have a sheet-like structure and a very small thickness compared with their lateral dimensions. Several 2D materials and their composites, including Graphene, MXenes, h-BN, carbon nitride, TMDs, molybdenum disulfide (MoS_2_), BP, and many others, are the research hot topics in recent times [1,2,11,12]. Graphene is a single layer of graphite and is one of the most commonly studied 2D materials. Its carbon atoms form a flat, hexagonal honeycomb pattern. Hexagonal boron nitride, or h-BN, is also called ‘white graphite.’ It has a similar honeycomb structure, but the carbon atoms are replaced by alternating boron and nitrogen atoms. h-BN looks like a white, slippery powder, and its flat, layered shape makes it a good lubricant. Transition Metal Dichalcogenides have a hexagonal lattice structure with threefold symmetry. MXenes are two-dimensional transition-metal carbides, nitrides, or carbonitrides commonly derived from layered MAX-phase precursors. The general formula of MAX phases is Mn+1AXn, where M represents an early transition metal, A is an element from groups 13 or 14, and X represents carbon and/or nitrogen. Selective etching of the A layers produces MXenes, which are generally represented by the formula Mn+1XnTx, where Tx denotes surface terminations such as –O, –OH, and –F. These surface terminations influence the surface chemistry and properties of MXenes. Table 1 highlights comparisons among these common 2D materials [1,2,11,12].

There are two major synthesis techniques for 2D materials: top-down and bottom-up approaches [1,3]. Top-down methods start with bulk layered materials and separate them into thinner nanosheets by overcoming the forces between adjacent layers. Mechanical exfoliation and liquid-phase exfoliation are some common examples of this method. For example, in the liquid-phase exfoliation process, mechanical forces such as sonication are applied to bulk materials dispersed in a suitable liquid medium to separate the layers. The liquid medium also helps stabilize the exfoliated nanosheets and prevents them from coming back together. In contrast, the bottom-up process, such as chemical vapor deposition (CVD), follows an additive approach where atoms or molecules come together step by step to produce the nanomaterials. One of the most popular bottom-up methods, CVD, is a unique fabrication technique where a solid material is deposited on a substrate from vapor-phase precursors through a chemical reaction. The choice of synthesis method can strongly influence the thickness, size, morphology, crystal structure, and surface properties of the resulting 2D material. Therefore, controlling the synthesis conditions is important for obtaining 2D nanomaterials with the desired properties and performance.

2D materials have many unique and useful properties, such as high ion conductivity, good mechanical flexibility, excellent electrical, optical, and thermal properties, and high endurance to stress. In addition, their surface chemistry can be modified relatively easily, which makes them useful for various applications. Due to these important features, the use of 2D materials is expanding at a remarkable rate. The global 2D materials market is projected to grow from USD 2.27 billion in 2020 to USD 2.86 billion by 2027, with a Compound Annual Growth Rate (CAGR) of 3.9% [13]. These features have made 2D materials useful for numerous applications, including energy storage, medical implants, sensing, drug delivery, biochemical sensing, environmental remediation, and many more. The combination of these properties makes 2D materials attractive for both existing and emerging technologies. Although 2D nanomaterials have attractive properties, they also face some challenges during synthesis and practical use. Their tendency to restack, aggregate, and lose some of their desirable surface properties can affect their overall performance. Therefore, different strategies are needed to control their structure, dispersion, and surface properties. Surfactants have emerged as one of the useful approaches to address these challenges.

## 3. Fundamentals of Surfactants

Surfactants (surface-active agents) are generally amphiphilic compounds containing hydrophilic and hydrophobic regions that enable them to interact with different phases and influence interfacial properties [8]. They are a diverse class of compounds widely used in a variety of applications because of their unique structure and physicochemical properties. They are widely used in industrial formulations and various research fields, including nanotechnology, drug delivery, energy storage, and environmental remediation [4].

In nanotechnology, surfactants are widely used to improve the dispersion and stability of nanomaterials. Many nanomaterials, such as graphene, carbon nanotubes (CNTs), TMDs, and black phosphorus, have hydrophobic surfaces. Organic solvents such as N-methyl-pyrrolidone and dimethylformamide can be used to disperse these nanomaterials; however, their high boiling points require high-temperature annealing for solvent removal. As a result, chemical degradation of the nanomaterials may occur, limiting their potential for biological applications. This limitation can be overcome by introducing amphiphilic surfactants into water together with the inorganic nanomaterials [4]. The global surfactants market was valued at USD 49.71 billion in 2025 and is projected to reach USD 77.25 billion by 2034 [14].

**Figure 2:**
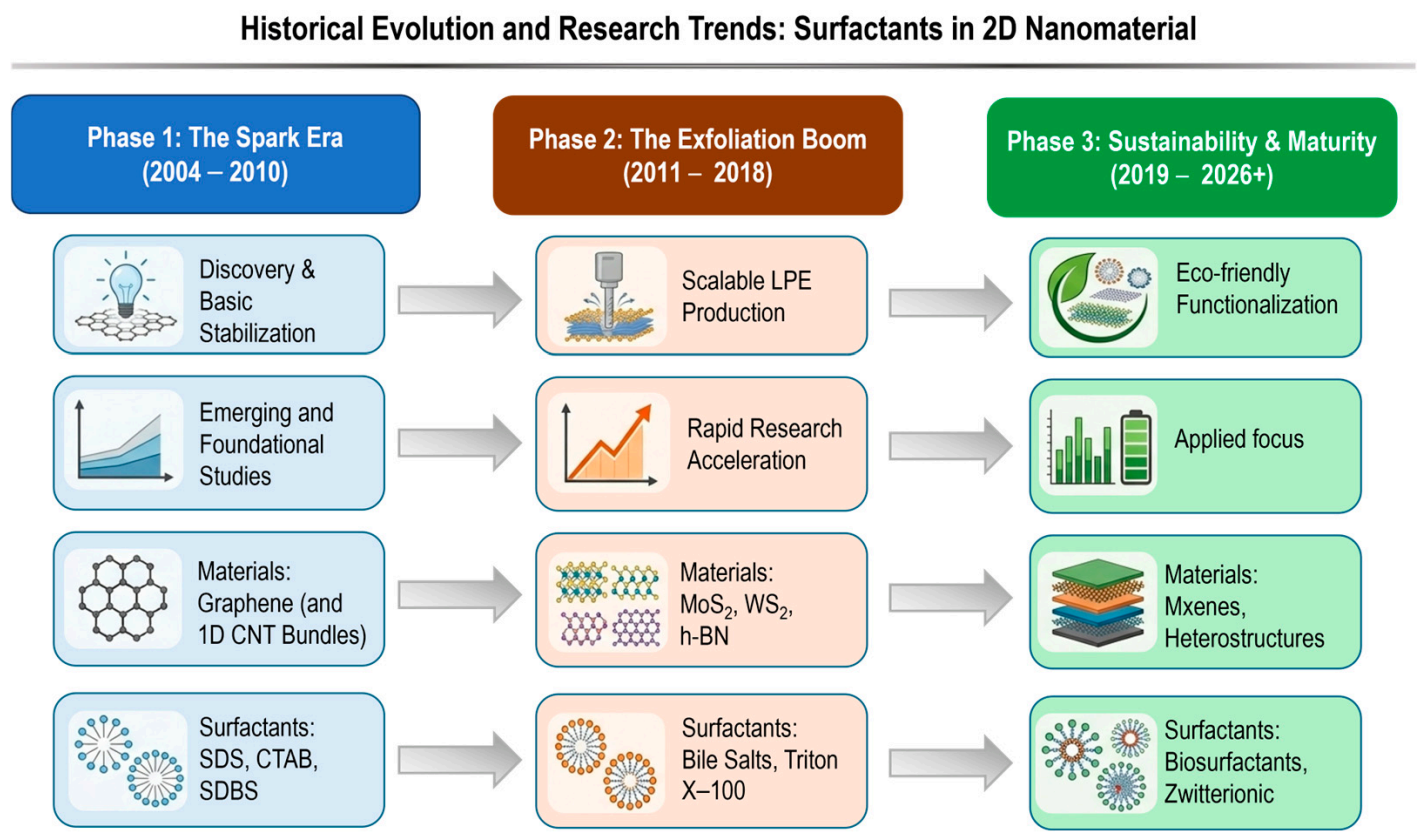
Qualitative historical overview and shifting research trends in surfactant-assisted processing for 2D nanomaterials.

Figure 2 provides a qualitative conceptual narrative of how surfactant-assisted processing of 2D materials has evolved through three distinct research paradigms. Phase 1 (2004–2010) represents the foundational spark of this field, where early liquid-phase exfoliation (LPE) models and aqueous stabilization parameters for pristine graphene sheets were directly adapted from the surface chemistry and dispersion mechanics originally optimized to debundle 1D CNT bundles in the early 2000s [15]; this cross-dimensional baseline provided the direct technical blueprint for successful early surfactant-assisted stabilization protocols in aqueous environments [16]. Phase 2 (2011–2018) marked an era of rapid material library expansion and structural scaling, during which the stabilization mechanics established for graphene were successfully extended beyond carbon chemistry to inorganic layered crystals like TMDs and h-BN [17], prompting researchers to expand the surfactant portfolio to include bile salts and non-ionic polymers to maximize monolayer extraction metrics [18] while transitioning toward industrial-scale high-shear processing methodologies [19]. Finally, Phase 3 (2019–2026+) reflects a distinct pivot toward targeted application engineering and eco-friendly processing parameters. In this modern era, contemporary research shifts focus away from sheer mass production yields toward optimizing precise interfacial constraints. This is evidenced by the development of standardized industrial ink formulation roadmaps with controlled rheology [20] and the non-destructive surface stabilization of chemically sensitive, non-carbon structures such as titanium carbide MXenes [21]. Concurrently, a growing sustainability and biocompatibility framework is driving the replacement of persistent, synthetic alkyl surfactants (such as SDS and CTAB) with degradable, bio-derived alternatives—ranging from simple industrial green surfactants like coco-glucoside [22] to complex natural macromolecules like human serum albumin (HSA) for clinical drug delivery platforms [23].

Surfactants can be classified in several ways depending on the criterion used. They may be categorized according to the source of the raw materials used for their production (renewable or non-renewable), their environmental impact, or their intended applications. From an environmental perspective, surfactants are often grouped as chemo-degradable, biodegradable, hardly degradable, or nondegradable. Finally, and perhaps most fundamentally, the most widely used classification is based on their chemical structure, particularly the nature of the hydrophilic head group, which largely governs their physicochemical properties and performance in different applications [24]. There are four types based on the charge of their head group: anionic, cationic, nonionic and amphoteric.

Anionic surfactants are the most common surfactants and carry a negative charge on the head group. Their hydrophobic part is usually an alkyl chain of varying length, alkylphenyl ether, or alkylbenzene, and their hydrophilic part is carboxyl, sulfate, sulfonate, or phosphate [8]. Examples include linear alkylbenzene sulfonate (LAS) and SDS. Common applications include nanomaterial exfoliation and dispersion.

Cationic surfactants have a positively charged head and their key property is strong antimicrobial activity. Common cationic surfactants include quaternary ammonium compounds (QACs), benzalkonium chloride (BAC), and CTAB. CTAB is also known as hexadecyltrimethylammonium bromide (HDTMA-Br); the two names refer to the same compound. The most common cationic surfactants are QACs. They consist of one or more hydrophobic hydrocarbon chains attached to a positively charged nitrogen atom, while the remaining positions are usually occupied by small alkyl groups, such as methyl or benzyl groups.

Nonionic surfactants do not carry a charge. Instead of ionizing in water, their solubility comes from their polar head groups. In general, the hydrophobic part is an alkylated phenol derivative, fatty acid, or long-chain linear alcohol, whereas the hydrophilic part is an ethylene oxide chain [8]. Nonionic surfactants have many advantages, such as low cost, high stability, low toxicity, and amphiphilic nature, and are popular in many industries, including the pharmaceutical industry [5]. Alkylphenol ethoxylate (APE) and alcohol ethoxylate (AE) are two common examples of this group.

**Figure 3:**
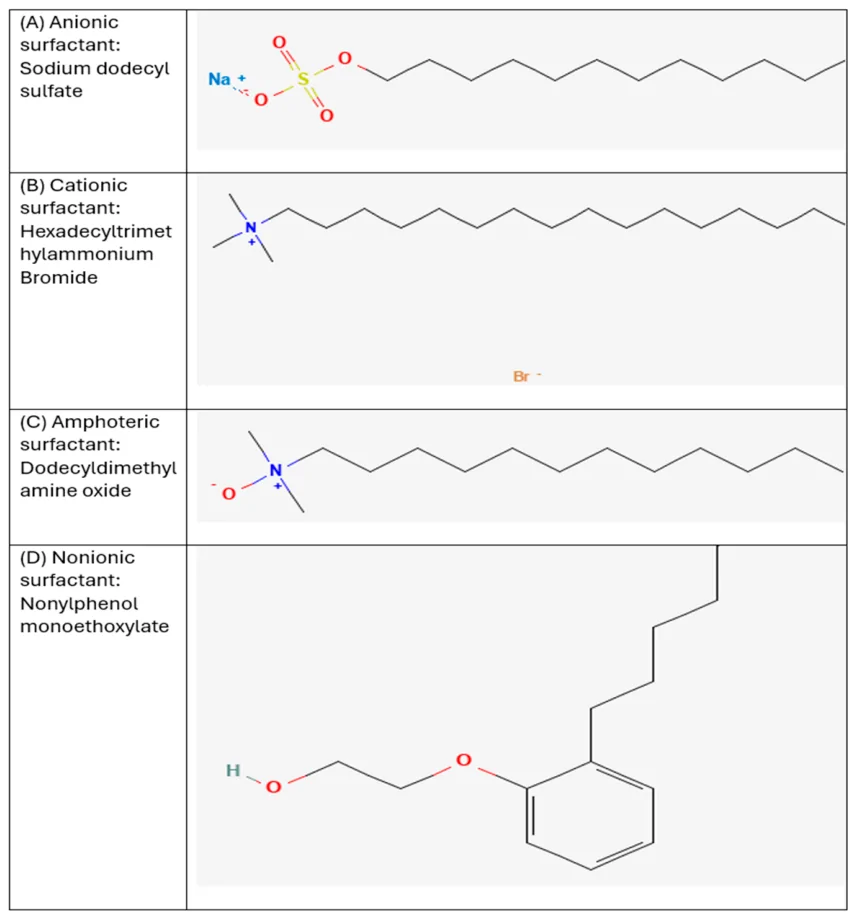
Chemical structures of representative surfactants from different classes: (**A**) anionic surfactant—sodium dodecyl sulfate, (**B**) cationic surfactant—Hexadecyltrimethylammonium Bromide, (**C**) amphoteric surfactant—Dodecyldimethylamine oxide, and (**D**) Nonionic surfactant—Nonylphenol monoethoxylate. Chemical structures were obtained from PubChem [25].

Amphoteric surfactants exhibit pH-dependent charge behavior and may change their net charge as the pH changes. Zwitterionic surfactants contain both positively and negatively charged groups within the same molecule and typically have a net charge of zero over a broad pH range. Although these terms are sometimes used interchangeably in the literature, they can be distinguished by their charge behavior under different pH conditions. Amine oxides (AOs), such as Dodecyldimethylamine oxide, are commonly classified as amphoteric surfactants, whereas Cocamidopropyl betaine is a representative zwitterionic surfactant. Representative structures of anionic, cationic, amphoteric, and nonionic surfactants are shown in Figure 3.

For clarity, this review distinguishes conventional surfactants from other additives used in the preparation and modification of 2D nanomaterials. Conventional surfactants are generally amphiphilic substances that adsorb at interfaces and influence interfacial properties. Other substances, including polymeric dispersants or stabilizers, biomolecular stabilizers, complexing agents, and synthesis additives, may also influence nanosheet dispersion, morphology, or growth but are not necessarily conventional surfactants. These materials are discussed according to their specific roles in the cited studies. Where a material such as graphene oxide is explicitly described as a macromolecular surfactant in the relevant literature, this terminology is retained with appropriate context.

Overall, with the unique structure and properties of the surfactants, they are a great candidate material in many research areas including nanotechnology and 2D materials. Their ability to reduce surface tension, improve dispersion, and stabilize interfaces has made them particularly valuable in this research area. Combining surfactants with 2D materials can be used to meet specific requirements in the synthesis, processing, and functionalization of nanomaterials. The next sections discuss how these characteristics have been utilized in the engineering of 2D nanomaterials for advanced applications.

## 4. Surfactant-Assisted Synthesis and Morphology Control of Two-Dimensional Nano Materials

Surfactants play multiple roles in the synthesis of 2D nanomaterials, ranging from regulating nucleation and directing crystal growth to controlling morphology and improving structural stability. Achieving precise control over the synthesis and morphology of 2D nanomaterials remains one of the major challenges in nanomaterials engineering. Among the various approaches developed to address these challenges, surfactant-assisted synthesis has become a central route to adjust the thickness, porosity, lateral dimensions, layer assembly, and overall morphology. However, surfactants do not operate through a single universal mechanism. Instead, their roles vary depending on the material system, synthesis route, and reaction environment.

Even though surfactants are commonly used in the synthesis of 2D nanomaterials, their roles go well beyond that of simply acting as dispersing or stabilizing agents. The way surfactant’s function depends on their molecular structure, the concentration at which they are used, and how they interact with the precursor species, enabling them to affect various stages of material formation such as precursor organization, nucleation, crystal growth, exfoliation, and post-synthesis stabilization. Mechanistically, surfactants influence 2D nanomaterial formation through several types of interactions, including electrostatic attraction or repulsion, surface adsorption, precursor coordination, interfacial confinement, and steric stabilization. The dominant mechanism depends on the chemical structure of the surfactant, the surface chemistry of the 2D material, and the synthesis conditions. Ionic surfactants can interact electrostatically with oppositely charged precursor species or material surfaces, thereby influencing precursor accumulation, nucleation, and molecular ordering at interfaces [9,26]. Surfactant molecules can also adsorb selectively onto growing crystal surfaces or specific crystal facets, modifying the relative growth rates of different planes and consequently controlling the morphology and thickness of the resulting material [6,27]. In some systems, interactions between surfactants and precursor species can further affect the organization and growth of the material [28]. After formation or exfoliation, surfactant adsorption can provide electrostatic or steric repulsion between nanosheets, reducing aggregation and restacking and thereby improving colloidal stability [29,30]. These interactions can also be influenced by surfactant concentration, surface coverage, solvent environment, and the specific surface characteristics of the 2D material. Therefore, the effect of a surfactant should not be considered universal; rather, its role depends on how its molecular structure and interfacial interactions modify the particular synthesis or processing environment. The major mechanisms and functions underlying surfactant-assisted synthesis and morphology control of 2D nanomaterials are discussed in this section.

### 4.1. Template-Directed Synthesis

Template-directed synthesis is a versatile technique that uses a pre-existing pattern or mold, called a template, to regulate the shape and structure of the nanomaterials. This approach provides a convenient way to control morphology during nanomaterial synthesis while enhancing stability. Templates are generally categorized as soft templates or hard templates. Common examples of soft templates include surfactant micelles, liquid crystals, and other self-assembled structures. In contrast, MOFs and solid-state surfactant crystals are commonly used as hard templates. For example, ref. [31] demonstrated a successful method to create ultra-thin nanoplates made of nickel sulfide and nickel selenide by using a nickel-based MOF (Ni-MOF) as a starting template. Here, the surfactant used, SDS, plays a shape-directing or structure-templating role during the formation of Ni-MOF precursors, leading to the successful formation of ultrathin layered structures. A different templating strategy was reported in [32], where sodium octadecyl sulfate (SOS) was employed as a solid-state surfactant template. In this route, metal ions are arranged in the interlayer space, then converted into isolated clusters by hydrolysis and condensation, and finally unfold those crystals into flat nanosheets. This is a significant discovery because it enables the synthesis of nonlayered and even amorphous nanosheets that are hard to obtain by conventional exfoliation techniques [32]. Similarly, ref. [33] also introduced an innovative solid-state templating mechanism in which the surfactant CTAB serves as a rigid framework to synthesize ultrathin platinum sheets. This study highlights that engineered solid-state surfactant crystals can work as both templates and regulators of precursor organization and nanosheet morphology to build custom-shaped platinum sheets that are highly efficient for next-generation electronic devices. Ref. [34] also supports the general concept of surfactant templating, in which amphiphilic surfactants self-assemble into organized structures that can guide material formation and morphology control.

### 4.2. Interface-Assisted Synthesis

All Interface-assisted synthesis is a chemical and physical fabrication approach in nanomanufacturing that confines the reaction to the boundary between two phases, such as solid–liquid, liquid–liquid, liquid–gas, or solid–solid interfaces. In this approach, surfactant molecules spontaneously self-assemble into monolayers or bilayers at the interface, creating highly organized reaction zones that accommodate precursor species for 2D growth. Surfactants play several important roles in this process by directing nucleation, regulating the distribution of precursor species, promoting molecular self-assembly and ordering, and assisting controlled crystal growth.

Ref. [9] is a review article on interface-assisted synthesis of 2D materials, where surfactant bilayers are highlighted as an important structure for creating reaction environments for ultrathin material production. The surfactant bilayers facilitate controlled synthesis by reducing structural defects. A more direct demonstration of the interface-assisted mechanism was reported in [26], where negatively charged surfactant monolayers (Sodium octadecyl sulfate monolayer) attracted positively charged intermediates through electrostatic interactions, increasing their local concentration and enhancing the molecular ordering. These findings indicate that the surface charge of the surfactant monolayer can influence precursor organization and the quality of the resulting films. Collectively, these studies are representative examples demonstrating the effectiveness of interface-assisted synthesis for engineering 2D nanomaterials through organized reaction environments instead of conventional physical templates.

### 4.3. Crystal Growth Regulation

Crystal growth regulation is one of the most important functions of surfactants during the synthesis of 2D nanomaterials. It refers to the deliberate control of nucleation and crystal development to achieve the desired size, shape, crystal phase, and overall morphology of the final material. Surfactants play an important role for regulating crystal growth by selectively interacting with growing crystal surfaces, modifying the growth kinetics, providing uniform growth environments, and reducing particle aggregation. For example, surfactants such as CTAB and SDS selectively adsorb onto specific crystal facets to block the growth of those facets and direct expansion along unblocked planes [27]. This allows researchers to control nanomaterial shapes including nanorods, and nanobelts. Being able to control these shapes is especially useful for things like catalysis, sensing, energy conversion, and other advanced technologies. Facet-selective adsorption should be distinguished from precursor complexation and intercalation, as these mechanisms involve different processes. Precursor complexation involves interactions between an additive and precursor species before or during nucleation, potentially influencing precursor availability and subsequent material formation. Facet-selective adsorption occurs when molecules preferentially bind to particular crystal surfaces and modify their growth rates. Intercalation, in contrast, involves the insertion of species into the interlayer spaces of a layered material. These mechanisms should not be treated as interchangeable, and a specific mechanism should be assigned only when supported by evidence from the corresponding study. An experimental example of additive-directed morphology control is reported in [28]. In this work, polyvinylpyrrolidone (PVP) was used during the synthesis of ultrathin MOF nanosheets where it played two important roles. It acted as a shape-directing agent by restricting vertical stacking and as a stabilizer after nanosheet formation. As a result, ultrathin nanosheets with well-controlled thickness and improved structural uniformity were successfully obtained. In addition, ref. [6] summarized several mechanisms by which surfactants regulate crystal growth of 2D materials. Selective facet adsorption, capping effects, oxidative etching, and structure-directing behavior are some of the mechanisms. These mechanisms demonstrate that surfactants play multiple roles during crystal growth, enabling control over the morphology and structural properties of 2D nanomaterials.

### 4.4. Morphology Control

To get the best properties and functions from 2D nanoparticles, it is important to control their thickness, size, aspect ratio, and crystal facets. The shape of a nanoparticle can influence how cells take it up, how well it works as a catalyst, and how it interacts with light. Surfactant-assisted synthesis methods help shape 2D materials by guiding how the building blocks come together, how crystals grow, and how surfaces interact. Surfactants can serve as stabilizers and templates that organize molecular intermediates [28,31,33,35,36]. Polymeric additives and ionic surfactants can influence nanosheet morphology through different mechanisms, including control of stacking, nucleation, and growth [28,31]. Surfactants such as CTAB and cetyltrimethylammonium chloride (CTAC) can help create ultrathin platinum sheets. Using solid surfactant crystals can produce nanosheets with clear rhombus or hexagonal shapes, and these sheets are extremely thin, usually between 1.5 and 3.0 nanometers thick [33]. The type of surfactant used in hydrothermal synthesis affects the shape of MoS_2_, leading to spherical, bulk-like, or flower-like structures. In the hydrothermal synthesis described in [36], polyethylene glycol (PEG) was used as an additive to control the morphology of the resulting material. The reported product consisted of uniformly dispersed spherical particles with slab lengths below 15 nm and fewer than six stacked layers. Similarly, coordinating additives such as Ethylenediaminetetraacetic acid (EDTA) can influence the crystal growth of MoS_2_ by inhibiting growth along the (00l) direction, resulting in porous flower-like structures, while dense spherical structures are formed without EDTA [7]. Another approach, described in [35], uses graphene oxide (GO) as a 2D macromolecular surfactant. GO can direct structure, control morphology and growth rates, and support defect and interface engineering, improving control over particle shape, size, and uniformity. While surfactants work through different mechanisms, they all aim to provide better control over the morphology of 2D nanomaterials.

### 4.5. Liquid-Phase Exfoliation

Liquid-phase exfoliation is a widely used top-down technique for making 2D nanomaterials from bulk layered materials. In this process, the bulk material is mixed into a liquid and broken down into nanosheets using external energy to separate the layers by overcoming the van der Waals forces between adjacent layers. During liquid-phase exfoliation, external mechanical forces such as sonication provide the energy needed to separate adjacent layers, while surfactants primarily help stabilize the newly exfoliated nanosheets and reduce their reaggregation, thereby improving dispersion stability and, in some systems, the overall exfoliation yield [30,37]. For example, sodium dodecylbenzene sulfonate (SDBS) can improve the dispersion stability of borophene during the liquid-phase exfoliation process [29]. This study showed that SDBS-assisted liquid-phase exfoliation produced thin borophene nanosheets with improved dispersion stability in an aqueous medium. The adsorption of SDBS on the borophene surface can help stabilize the exfoliated nanosheets and reduce their reaggregation, although the specific molecular mechanism responsible for the exfoliation process is not fully established. Overall, the study demonstrated that SDBS can support the formation of stable borophene dispersions during liquid-phase exfoliation. Another experimental demonstration of surfactant-assisted LPE is reported in [37], where SDBS plays an effective role in the exfoliation of the layered silicate material Egyptian blue (EB) into ultrathin nanosheets. The researchers applied a combined method of planetary ball milling, SDBS-assisted bath sonication, and centrifugation to produce well-dispersed, uniform nanosheets effectively. Ref. [37] also compared surfactant-assisted exfoliation vs. plain solvent-assisted exfoliation. They tried the same procedure with plain water, several organic solvents such as propanol, dimethyl sulfoxide (DMSO), etc., and two surfactant solutions (SDS and SDBS). The experiment gives much better outcomes with the surfactant-assisted option. These results show that the effectiveness of liquid-phase exfoliation strongly depends on the choice of surfactant and solvent system.

Surfactant concentration and solution chemistry influence both nanosheet production and colloidal stability. In liquid-phase exfoliation of WS_2_, Griffin et al. [30] showed that the optimum surfactant concentration was not necessarily linked to the critical micelle concentration (CMC), highlighting the need to distinguish exfoliation from subsequent stabilization. In aqueous dispersions of MoS_2_, WS_2_, and MoSe_2_, Abreu et al. [38] showed that surfactant identity and concentration influence dispersibility and that pH and zeta-potential measurements provide insight into the resulting dispersion behavior. In CTAB-stabilized MoS_2_ dispersions, Gupta and Vasudevan [39] demonstrated that ionic strength affects zeta potential and electrostatic stabilization. These findings indicate that surfactant concentration and solution conditions should be optimized for the specific surfactant–material system rather than assuming that concentrations above or below the CMC will always improve exfoliation or stability.

### 4.6. Dispersion and Stabilization

Nanomaterials have a natural tendency to restack or aggregate due to their high surface energy and large surface area. Therefore, dispersion and stabilization are very important for preserving the unique properties of 2D nanomaterials during synthesis and processing. Surfactants act like a protective shield by adsorbing onto the surfaces of separate layers and preventing their aggregation. This protection typically works in two ways: electrostatic repulsion, where the electrical charges of ionic surfactants push the sheets away from each other, and steric hindrance, where adsorbed polymeric additives can create a physical barrier between the sheets. For example, ionic surfactants such as SDBS can provide electrostatic stabilization of exfoliated nanosheets, while polymeric additives such as PVP can provide steric stabilization after nanosheet formation [28,29]. Similarly, GO acts as a 2D surfactant, helping particles spread out evenly and reducing clumping through steric stabilization and interactions at the surface [35]. These examples show that, although different surfactants use different stabilization mechanisms, they all contribute to maintaining well-dispersed and structurally stable 2D nanomaterials suitable for further processing and practical applications. Surface coverage also influences the effectiveness of surfactant-assisted stabilization. Adsorption of CTAB onto MoS_2_ surfaces affects their surface charge and zeta potential, which are relevant to electrostatic interactions between nanosheets [39]. More generally, incomplete or nonuniform surfactant coverage may leave regions less protected against aggregation, whereas adsorbed surfactant layers can provide electrostatic or steric barriers to nanosheet association. However, the effects of surface coverage depend on the surfactant–material system and should not be assumed to be identical across all 2D nanomaterials.

A summary of the major mechanisms and functions discussed above is provided in Table 2.

## 5. Applications of Surfactant-Engineered 2D Nanomaterials

Surfactant-assisted engineering can enhance the properties of 2D materials by controlling morphology, dispersion, and surface chemistry. The extent to which these changes are beneficial will depend on the kind of surfactant used and the method of modification, since they can lead to better interactions between 2D materials and biological systems, improve the materials’ electrochemical performance, or increase their ability to interact with and remove pollutants. The following section discusses the applications of surfactant-assisted 2D nanomaterials in drug delivery, energy storage, and environmental remediation.

### 5.1. Drug Delivery

In drug-delivery applications, surfactants play an important role in the preparation and stabilization of 2D nanomaterials. Materials such as graphene and GO, TMDs, BP, LDHs (Layered double hydroxides), h-BN, and MXenes have attracted considerable interest because of their large surface area and ability to accommodate therapeutic molecules. However, aggregation and restacking of nanosheets can reduce the accessible surface area and limit their effectiveness as drug carriers [23,40]. Surfactants are commonly used during liquid-phase exfoliation and synthesis to improve nanosheet dispersion and control their structural properties. Ionic surfactants, including SDS, SC, and CTAB, and nonionic surfactants, such as Tween 80, Triton X-100, and Pluronic F127, can adsorb onto nanosheet surfaces and help prevent aggregation through electrostatic or steric stabilization, depending on the surfactant and material involved [40]. These interactions can improve aqueous dispersibility and colloidal stability while also influencing the surface area available for drug loading and subsequent functionalization [18,41]. However, improvements in drug-delivery performance may also result from targeting ligands, the intrinsic properties of the 2D material, or additional surface modifications. It is therefore important to distinguish the contribution of surfactants from these other factors. The following discussion focuses on the roles of specific surfactants in nanosheet preparation, stabilization, and drug delivery, with attention to the experimental comparisons and reported improvements where such information is available.

Although surfactant modification has been reported to improve the aqueous dispersibility and colloidal stability of 2D nanosheets [41], its direct contribution to drug-loading efficiency and cellular interactions remains less clearly established. In many studies, surfactant-assisted preparation is followed by additional functionalization, making it difficult to distinguish the effects of surfactant stabilization from those of other surface modifications. Therefore, comparisons between surfactant-modified and unmodified nanosheets are important for evaluating their specific contributions to drug-delivery performance [18].

The importance of surfactant adsorption is particularly evident during liquid-phase exfoliation. Surfactant adsorption can help stabilize newly exposed nanosheet surfaces and limit reaggregation after exfoliation. Although surfactant adsorption may influence interfacial interactions, its direct role in weakening Van Der Waals attraction between adjacent layers depends on the material and processing conditions. For ionic surfactant-stabilized WS_2_ nanosheets, both mass yield and lateral dimensions decreased sharply at surfactant concentrations above approximately 10 mM, whereas stable dispersions were obtained at concentrations as low as 0.07 mM, with absolute zeta potentials exceeding 40 mV [30]. HSA provides an example of a biomolecular stabilizer used in the exfoliation and stabilization of BP nanosheets. Its adsorption on the BP surface has been reported to improve aqueous dispersibility and colloidal stability. In addition to assisting nanosheet preparation, HSA provided an interface for loading paclitaxel (PTX) through hydrophobic interactions. The resulting BP-HSA-PTX formulation was investigated for chemotherapy combined with near-infrared (NIR)-induced photothermal treatment [42]. Thus, the same biomolecular component contributes to exfoliation, stabilization, and preparation of the nanosheet surface for subsequent drug loading. However, the enhanced therapeutic response under NIR irradiation reflects the combined effects of the drug formulation and BP photothermal activity rather than an independently established effect of HSA alone.

GO and reduced graphene oxide (rGO) are among the most widely investigated 2D materials for cancer-related applications [23]. Their planar structures provide a large accessible surface, while the chemical groups present on GO offer numerous possibilities for attaching drugs and targeting molecules. However, maintaining stable nanosheet dispersions is important during drug loading and formulation, as aggregation can reduce the accessible surface area. Surfactants can help address this limitation by stabilizing GO nanosheets through electrostatic or steric interactions, depending on their surface chemistry [41]. Methotrexate-loaded, folic acid-functionalized GO has been investigated as a targeted anticancer delivery system [43]. However, the reported therapeutic effects do not independently establish a surfactant-specific contribution. These improvements are mainly associated with the drug-carrier formulation and folic acid functionalization rather than a demonstrated surfactant-specific effect. Therefore, although surfactants may contribute to the preparation and stability of GO-based drug carriers, their specific effects on drug-loading efficiency and therapeutic performance need to be established through appropriate experimental comparisons.

BP nanosheets represent another important class of 2D materials for biomedical delivery. Unlike many persistent inorganic nanomaterials, BP can undergo degradation under physiological conditions, which has attracted considerable interest in applications where long-term material accumulation is undesirable [40]. Their large surface area and optical properties have supported investigations of drug delivery and externally triggered therapeutic applications. Surfactant-mediated stabilization is important because BP is chemically reactive and its dispersion behavior can strongly influence its performance in biological environments. Biomolecular or biocompatible surface coatings can therefore serve two purposes: protecting and stabilizing the nanosheets during formulation while providing an interface through which drugs or biological molecules can be incorporated [42]. However, the biodegradability and photothermal response of BP are primarily material-related properties, whereas improvements in dispersion stability and drug incorporation depend on the particular surface treatment. These material-related and surface modification effects should be distinguished when evaluating BP-based drug-delivery systems.

MXenes have expanded the range of 2D materials being considered for biomedical drug delivery. Their large accessible surface area, hydrophilic character, high electrical conductivity, and efficient photothermal conversion provide several characteristics that are useful for multifunctional therapeutic platforms [44]. Although MXenes were initially studied extensively for electrochemical energy-storage applications, their surface chemistry has subsequently generated considerable interest in biomedical research. Appropriate surface engineering allows therapeutic molecules to be incorporated while improving the compatibility of the nanosheets with physiological environments. Functionalization using PEG, PVP, Pluronic surfactants, and phospholipid-based coatings has been investigated as a means of improving colloidal stability, extending circulation, and limiting nonspecific protein interactions. For example, Liu et al. developed a surface-modified Ti_3_C_2_T_x_ MXene system incorporating gold nanoparticles, PEG, and doxorubicin [45]. In this formulation, PEG was used for surface modification, while gold nanoparticle incorporation contributed to the photothermal properties of the resulting platform. The system also exhibited pH- and NIR-responsive drug release. These findings demonstrate the potential of combining surface modification with other functional components, although the individual effects of PEG coating, gold nanoparticles, and the MXene structure should be distinguished when interpreting the reported therapeutic performance.

Surfactant-assisted synthesis has been used to prepare ultrathin porphyrinic 2D MOF nanosheets, including Cu–TCPP(Fe), with PVP helping to restrict layer stacking and stabilize the resulting nanosheets. The structural properties and tunable porosity of 2D MOF nanosheets also make them promising platforms for drug delivery and photodynamic therapy [46]. Rather than releasing therapeutic molecules solely via passive diffusion, engineered 2D nanocarriers can be designed to respond to specific conditions in their biological environment. Endogenous triggers include acidic pH, increased glutathione concentrations, reactive oxygen species (ROS), and enzyme activity, whereas externally applied stimuli can include near-infrared (NIR) irradiation, magnetic fields, ultrasound, and electrical stimulation [47]. Surface functionalization further expands these possibilities. Following stabilization of the nanosheets, antibodies, peptides, aptamers, folic acid, hyaluronic acid, or transferrin can be introduced to recognize receptors that are overexpressed by cancer cells [48]. Combining such targeting approaches with chemotherapeutic drugs, imaging agents, photosensitizers, or photothermal components allows the development of multifunctional systems in which localization, therapeutic release, and treatment can be coordinated within a single platform. Table 3 introduces the role of surfactants in drug delivery for 2D nanomaterials.

Despite the progress achieved with surfactant-engineered 2D nanomaterials, their translation into clinically applicable drug-delivery systems still requires several issues to be resolved. Long-term toxicity, metabolic degradation, biodistribution, and clearance need careful investigation through well designed in vivo studies [51]. Attention should also be given to the surfactant itself. A molecule that performs effectively during exfoliation or synthesis may remain partially associated with the nanosheet surface, and its biological effects therefore need to be considered together with those of the underlying 2D material. Reproducible manufacturing represents another major challenge. Parameters such as nanosheet thickness, lateral dimensions, surface chemistry, surfactant coverage, and drug-loading capacity need to remain consistent from batch to batch if these systems are to progress beyond laboratory-scale studies. Large-scale synthesis must also preserve the structural characteristics responsible for their therapeutic performance. Future studies should therefore place greater emphasis on environmentally compatible and biodegradable surfactants, scalable preparation procedures, standardized physicochemical characterization, and systematic toxicity assessment. Multifunctional platforms that integrate targeted delivery with imaging, biosensing, and combination therapy are likely to remain an important research direction. More importantly, establishing clear relationships between surfactant chemistry, nanosheet structure, biological interactions, and therapeutic response will be necessary to guide the rational design of clinically relevant surfactant-engineered 2D drug-delivery systems. The toxicity of the resulting formulation may depend on the surfactant’s chemical structure and concentration, as well as its interactions with the 2D material. Therefore, biocompatibility should be evaluated for the complete formulation rather than inferred from the properties of its individual components [40]. Table 3 represents the summary of surfactant-engineered 2D nanomaterials for drug delivery discussed in this section.

### 5.2. Energy Storage

2D nanomaterials have received considerable attention for electrochemical energy storage (EES), including lithium-ion batteries (LIBs), sodium-ion batteries (SIBs), supercapacitors, and hybrid energy-storage devices. Their large specific surface area, short ion-diffusion pathways, tunable surface chemistry, and, for many 2D materials, favorable electrical properties provide several advantages for electrochemical applications. Graphene, transition-metal oxides (TMOs), TMDs, and MXenes are among the materials investigated for this purpose [52]. However, aggregation and restacking of nanosheets through van der Waals interactions can decrease the accessible surface area, restrict ion transport, and affect structural stability during repeated charge–discharge cycles. Surfactants can help address these limitations during material synthesis and processing by functioning as stabilizers, structure-directing agents, and surface modifiers.

Liquid-phase exfoliation provides a practical route for preparing 2D nanosheets while retaining much of the structural character of the parent layered material. This approach has been applied to prepare MoS_2_ nanosheets for sodium-ion battery electrodes [53]. During liquid-phase processing, surfactant adsorption can help stabilize newly exposed MoS_2_ surfaces and limit reaggregation after mechanical separation. Although adsorption may influence interfacial interactions, direct weakening of the interlayer attraction should not be assumed without specific supporting evidence. Maintaining separated MoS_2_ sheets is beneficial for ion storage because the accessible interlayer regions provide pathways for Na^+^ transport and increase the surface available for electrochemical reactions. The choice of surfactants can also influence the morphology produced during synthesis. Ionic surfactants, including CTAB, SDS, and SC, as well as Gemini surfactants and block copolymers, have been used to regulate nanosheet growth, dispersion, and hierarchical organization [54]. Their function therefore extends beyond stabilization; depending on the synthesis route, surfactants can influence how the active material is assembled and ultimately how readily ions can access the electrode structure. However, improvements in sodium-ion storage performance should not be attributed solely to surfactant-assisted exfoliation unless the study directly compares surfactant-treated and untreated MoS_2_ electrodes. Such comparisons are necessary to distinguish the effects of surfactant stabilization from those associated with reduced nanosheet thickness and changes in electrode morphology.

MXenes provide another important example in which surface and interfacial chemistry are closely connected to energy storage behavior. Their metallic conductivity, hydrophilic character, and layered structures make them suitable for electrochemical systems that require rapid transport of both electrons and ions. An important feature of MXenes is the presence of surface terminations, commonly –O, –OH, and –F, which can substantially alter ion adsorption, intercalation behavior, charge transfer, and theoretical storage capacity [55,56]. Consequently, considerable effort has been directed toward controlling MXene surfaces and preventing adjacent sheets from forming densely restacked structures [57]. Strategies such as interfacial modification, polymer incorporation, and hybridization with graphene, carbon nanotubes, or metal oxides have been investigated to maintain accessible interlayer regions and improve the conductive network of the electrode [58]. These structural and chemical modifications have allowed MXene-based materials to be investigated across lithium-ion, sodium-ion, and potassium-ion batteries as well as supercapacitors [59]. Their performance, however, depends not simply on the intrinsic conductivity of the MXene but also on how effectively the surface terminations, interlayer distance, and electrode architecture are controlled during material processing. These improvements should not be considered direct surfactant effects unless a surfactant is specifically involved in the preparation process and its contribution is experimentally demonstrated.

The control of interlayer organization becomes particularly important for sodium-ion and other emerging ion-storage systems because the size and transport behavior of the charge-carrying ions differ from those encountered in conventional lithium-ion batteries. Surfactant-assisted liquid-phase processing can help maintain layered materials such as BP and MoS_2_ as stable nanosheet dispersions, providing greater access to the surfaces and interlayer regions involved in ion transport. Ion transport is also influenced by the composition and surface chemistry of 2D materials. For example, first-principles calculations have shown that vanadium doping can improve lithium adsorption and diffusion behavior in Ti_2_CO_2_ Mxene [60]. Hybridization offers another means of preserving these pathways. For example, MXene–rGO architectures can reduce dense restacking of MXene layers while establishing an interconnected conductive framework. The more open lamellar arrangement provides additional space for ion transport and can better accommodate structural changes during repeated insertion and extraction of relatively large ions. MXene-based hybrid structures have been investigated for electrochemical energy storage, with their potential benefits associated with improved electrode architecture, electrical connectivity, and ion accessibility [61]. These studies indicate that controlling nanosheet separation and interfacial organization is important for non-lithium-ion storage. However, the improved electrochemical performance of MXene–rGO architectures is primarily associated with hybridization and the resulting electrode structure rather than an independently demonstrated surfactant effect.

Surfactant-assisted liquid-phase exfoliation is also useful for preparing layered materials for electrochemical applications. During exfoliation, surfactant molecules adsorb on newly exposed surfaces and help keep the separated nanosheets dispersed. SC, for example, has been used for liquid-phase exfoliation of h-BN in aqueous media, producing few-layer nanosheets for electrochemical investigations [62]. An important point, however, is that the surfactant may affect more than the exfoliation process itself. Molecules remaining on the nanosheet or electrode surface can alter interfacial interactions and may contribute to the measured electrochemical response. Consequently, an increase in capacitance after surfactant-assisted exfoliation should not automatically be attributed only to a decrease in nanosheet thickness. Appropriate control experiments are needed to separate the contribution of the exfoliated material from that associated with residual or adsorbed surfactant. This consideration is particularly relevant when comparing surfactant-exfoliated nanosheets with their bulk counterparts. Although liquid-phase exfoliation provides an attractive route to thin, solution-processable 2D materials, the surfactant concentration, nanosheet dimensions, and purification procedure must be carefully controlled if reproducible electrode performance is to be achieved. Experimental evidence demonstrates that residual surfactants can influence the electrical performance of 2D-material electrodes. For example, thermal treatment at approximately 320 °C was reported to increase the electrical conductivity of coco-glucoside-stabilized graphene films by about 15-fold, whereas films prepared using SC and SDBS showed only marginal conductivity changes under the reported conditions [22]. The study associated this difference with the decomposition behavior of the surfactants and the persistence of residues from the other surfactants. However, thermal treatment may also alter inter-sheet contacts and other structural characteristics, so the conductivity changes should not be attributed solely to surfactant removal. This example highlights the importance of evaluating residual surfactants alongside electrode structure and electrochemical performance.

Another use of surfactants is to control how nanoscale components are organized during electrode fabrication. Amphiphilic surfactants and block copolymers can guide inorganic precursors or nanoparticles into ordered structures through cooperative self-assembly. This provides a way to adjust pore dimensions, nanosheet thickness, and the arrangement of individual building blocks during synthesis. Such structural control is particularly relevant to ordered mesoporous 2D materials because an open pore network can improve electrolyte access while shortening the distance required for ion transport. Porous architecture may also provide additional space to accommodate structural changes during repeated ion insertion and extraction. Wei et al. reviewed how surfactant-assisted cooperative self-assembly can organize nanoparticles into well-defined nanostructures by controlling interactions at the nanoscale [63]. In these systems, the surfactant primarily serves as a structure-directing component during assembly and can subsequently be removed. If the organized architecture is retained after removal, interconnected pores and exposed surfaces become available for electrochemical reactions. This type of structural design is relevant to batteries and supercapacitors, where electrolyte accessibility, short transport pathways, and structural stability strongly influence electrode performance [52,63].

Despite these advantages, several practical issues associated with surfactant-assisted processing still need to be addressed. One concerns the surfactant remaining after synthesis. Excess organic material on an electrode surface may interfere with charge transport or reduce access to electrochemically active sites, whereas complete removal may not always be desirable when the surfactant contributes to nanosheet stabilization or to the preservation of a particular architecture. The required degree of surfactant removal therefore depends on both the material and the purpose for which the surfactant was introduced. Reproducibility is another concern, particularly when a synthesis method is transferred from laboratory-scale preparation to larger batches. Parameters such as nanosheet thickness, lateral dimensions, surface chemistry, interlayer spacing, and surface coverage need to be controlled consistently because each can influence electrochemical behavior [52,58,59]. Future studies should also consider surfactants that are easier to remove, have lower environmental impact, and maintain electrode performance after processing. Experimental studies combined with computational analysis may help clarify these relationships and guide the selection of surfactants for particular electrode materials. Overall, surfactants can contribute to 2D energy-storage materials at several stages, including exfoliation, dispersion, structural organization, and interface formation. Understanding these individual contributions will be important for designing 2D electrodes with reproducible performance in batteries, supercapacitors, and flexible energy-storage devices.

### 5.3. Environmental Remediation

Rapid global industrialization and urbanization have caused a significant increase in environmental pollution, which has become a major concern in modern times. Pollutants like dyes, heavy metals, gases, pharmaceuticals, and other toxic substances contaminate the atmosphere, putting both people and ecosystems at risk. According to the World Health Organization (WHO), in 2021, household and outdoor air pollution caused an estimated 6.6 million deaths and 184 million DALYs (Disability-Adjusted Life Year) worldwide, mainly due to respiratory and cardiovascular diseases, lung cancer, and stroke [64]. It is also alarming that around 99% of people worldwide live in places where air pollution exceeds WHO guidelines [64]. 2D nanomaterials can contribute to addressing many environmental concerns, such as pollutant adsorption, due to their distinctive physical, optical, and chemical properties, including large surface area, tunable surface chemistry, good dispersion, and high aspect ratio. At the same time, 2D materials have some limitations, particularly their tendency to restack due to interlayer interactions. Using surfactants with 2D materials can help solve these issues by acting as shape-directing agents and functional coatings. With surfactants, 2D materials become more versatile and effective for tasks like photocatalytic degradation, pollutant adsorption, and oil/water separation [65,66,67,68,69]. This section highlights key examples of how surfactants improve the environmental cleanup abilities of 2D nanomaterials.

Photocatalytic degradation uses light and a photocatalyst to transform pollutants into intermediate products and, potentially, simpler compounds. However, decolorization or disappearance of the parent pollutant does not necessarily indicate complete mineralization or reduced toxicity, as these are distinct outcomes that require separate assessment. This process relies on light-activated semiconductor catalysts, whose composition, morphology, thickness, and surface area can influence photocatalytic performance. This method is widely used for removing dyes and other organic pollutants from contaminated water. Surfactants can assist in the synthesis and morphology control of 2D materials, which can ultimately affect photocatalytic activity. Mishra et al. demonstrated that simply sonicating bulk Tungsten disulfide (WS_2_) and MoS_2_ in dilute aqueous household detergent was sufficient to exfoliate the bulk materials into few-layer nanosheets. The detergent helped lower the surface tension of water and allowed ultrasonic energy to effectively peel layers off the bulk material and disperse them. These surfactant-exfoliated WS_2_ and MoS_2_ nanosheets exhibited exemplary performance by degrading over 90% of industrial pollutant Brilliant Green dye [65]. A similar connection between surfactant-assisted preparation and photocatalytic performance can be observed in [66]. In this study, the researchers compared four additives-urea, CTAB, PVP, and oxalic acid-during the hydrothermal synthesis of MoS_2_ [66]. The resulting materials exhibited different morphologies and optical properties. Among the tested samples, urea-assisted MoS_2_ showed the highest reported photocatalytic degradation efficiency (about 59%) for industrial wastewater. This example illustrates how different synthesis additives can influence material properties and photocatalytic performance, although the observed effects should not be attributed to conventional surfactant action for all additives. Also, combining two light-absorbing materials can produce a hybrid photocatalyst with improved properties for environmental remediation. Ho et al. in [70] combined graphitic carbon nitride (g-C_3_N_4_) with porphyrin molecules using CTAB, which helped organize the porphyrin molecules into uniform nanorods through self-assembly. The resulting hybrid material absorbed light across a broad range of the visible spectrum. Under simulated sunlight irradiation, the hybrid material achieved an approximately 96% decrease in Cr(VI) concentration within 30 min and nearly 100% methylene blue removal after 100 min. The authors proposed that photogenerated electrons reduce Cr(VI) to Cr(III). However, pollutant degradation does not necessarily imply complete mineralization or reduced toxicity.

All these cases show that surfactants or synthesis additives serve functions beyond facilitating 2D material formation. They also influence the material’s morphology, surface area, and dispersion, which in turn affect its interactions with pollutants during photocatalytic treatment. However, a decrease in pollutant concentration does not necessarily indicate complete mineralization or reduced toxicity, and these outcomes require confirmation through appropriate chemical analyses and toxicity assessments. Surfactant assistance helps 2D materials adsorb organic pollutants mainly by changing how the sheets are dispersed, stacked, and chemically presented to the pollutant. Adsorption is a commonly used approach to remove organic pollutants from contaminated water. Surfactant-assisted 2D nanomaterials are useful for this purpose as they adjust the surface properties of the nanomaterial and improve the interaction with pollutants. The effect can be seen with different materials and surfactants. Organic dyes are widely used in many industries, such as textiles, paper, cosmetics, food, etc. Thus, effectively treating industrial wastewater is a major environmental concern. Surfactant-assisted graphene is a good example of this approach, as it can rapidly adsorb dyes due to its good dispersion. Ref. [67] explored graphene dispersions stabilized by anionic (SDS), cationic (CTAB), and non-ionic (Pluronic F108) surfactants to treat solutions of methylene blue (MB) and methyl red (MR). The maximum adsorption capacity was achieved with SDS-exfoliated graphene, reaching 782.3 mg/g at 25 °C. The adsorption-isotherm experiments used initial MB concentrations of 5–150 ppm at pH 7. The original study defines adsorption capacity relative to the adsorbent mass but does not explicitly clarify whether this mass refers to graphene alone or to the SDS-exfoliated graphene material, including its adsorbed surfactant. The removal mechanism can involve a combination of electrostatic attraction between the surfactant-modified graphene surface and the dye molecules. For example, the toxic anionic dye methyl orange (MO) can be removed from textile wastewater using CTAB-modified Ti_3_C_2_T_x_ MXene [71]. In this process, the positively charged CTAB coating interacts with the negatively charged dye, while hydrogen bonding and π-cation interactions also contribute to the adsorption process. CTAB modification increases the interlayer spacing of MXene and provides more accessible sites for dye adsorption. This CTAB-modified MXene, called CMM, is a practical and reusable option for cleaning dye-contaminated water. Removing different types of pollutants from the same water source can be challenging because they may have very different chemical properties.

Surfactant functionalization provides a useful approach to address this problem by introducing different interactions on the surface of 2D nanomaterials. A GO sponge modified with the cationic surfactant HDTMA-Br can reduce the repulsive forces between sheets and, in turn, promote the formation of a more stable three-dimensional structure. This unique structure enabled the simultaneous adsorption of Cu^2+^ and bisphenol A (BPA) from water, with Langmuir-model maximum adsorption capacities of 59.7 and 141.0 mg/g, respectively, at 25 °C and pH 5.0. These capacities are reported per gram of the GO/HDTMA composite adsorbent. The adsorption-isotherm experiments used initial Cu^2+^ and BPA concentrations of 5–100 mg/L [72].

The problem of oil and water separation resulting from industrial waste and environmental spills remains a long-term environmental issue. To achieve this, controlling surface wettability to allow water or oil to pass through the surface is important; therefore, surfactants can play a significant role by controlling the surface chemistry of 2D materials and improving the wettability of the resulting material. A notable method is described in [68], in which the researchers coated metal meshes with GO, a widely used 2D material, with the cationic surfactant CTAB. This modification endows the mesh with a surface that can be switched between two states: hydrophilic (water-loving, oil-repelling) and superhydrophobic (oil-loving, water-repelling)-depending on the surface treatment applied. As a result, the material achieved high separation efficiency, making it a promising solution for cleaning up oily wastewater. On the same note, ref. [73] discusses the use of MXene-based 2D materials for oil–water separation. The hydrophilic surface of MXenes can promote water transport while repelling oil, and surface modification can further adjust the wettability of the material for different separation conditions. The review also mentions that surfactant chemistry can influence the charge of oil droplets and, as a result, affect their interaction with the MXene surface. The environmental suitability of surfactant-assisted 2D nanomaterials depends not only on pollutant-removal performance but also on surfactant toxicity, biodegradability, and the potential release of surfactants and their transformation products during treatment and disposal [74]. For example, Ge et al. reported a 96 h EC_50_ of 145 ± 13.35 µg/L for CTAC based on growth inhibition in Chlorella vulgaris, highlighting the importance of evaluating surfactant toxicity to aquatic organisms [75]. Where technically feasible, biodegradable or bio-derived surfactants may offer alternatives; however, their environmental safety and treatment performance should be evaluated rather than assumed. Future studies should quantify surfactant leaching and degradation under realistic treatment conditions and assess the environmental effects of released surfactants and their transformation products.

Ref. [69] also highlights the growing interest in 2D MOFs for a wide range of environmental remediation applications, e.g., removing pollutants from water, photocatalytic degradation and membrane separation. The review also noted the role of surfactants such as SDS, CTAB, and PVP, which can interact with specific crystal planes and help produce thinner 2D structures with desirable properties. Despite their promising performance, practical applications of surfactant-assisted 2D nanomaterials require consideration of the recovery, regeneration, and reuse of the materials. The potential release of residual surfactants or nanosheets into treated water should also be evaluated to minimize secondary contamination. Further studies should evaluate material stability and performance over repeated treatment cycles.

**Figure 4:**
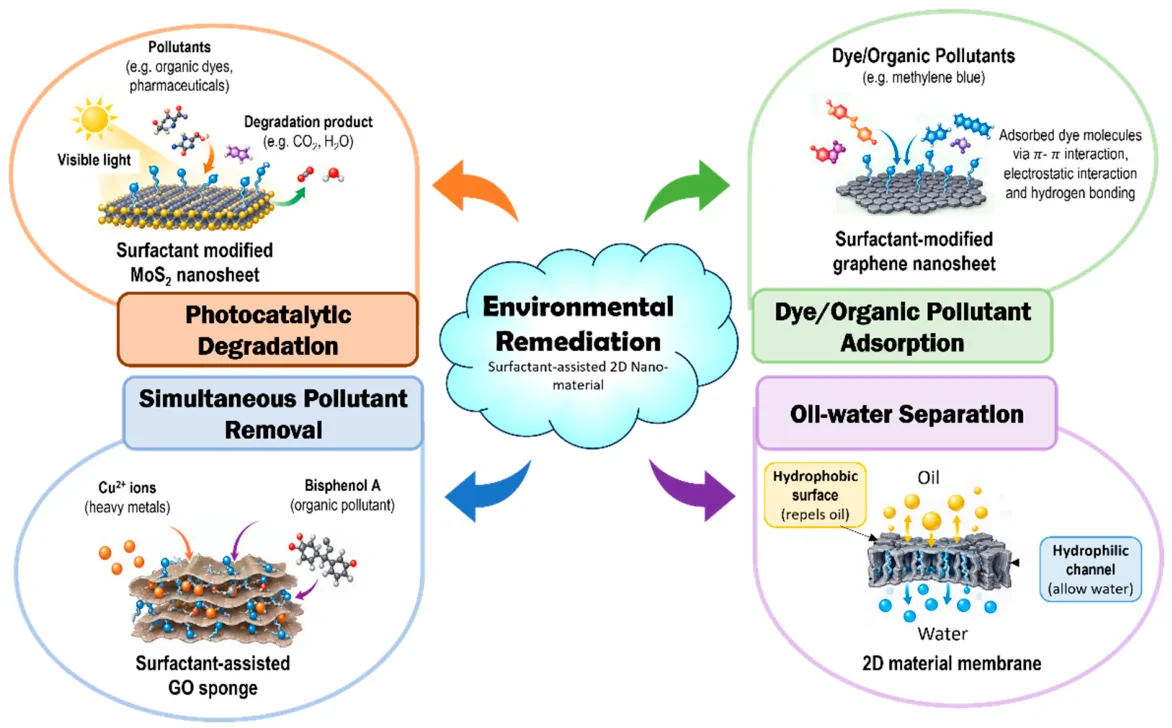
Functional application pathways of surfactant-assisted 2D nanomaterials in environmental remediation.

Overall, surfactant-assisted 2D materials have emerged as promising candidates for environmental remediation through different approaches, including pollutant degradation, adsorption, oil/water separation, and others as illustrated in Figure 4. The key idea is that surfactants can improve morphology, interfacial behavior, and surface properties of 2D materials, which can enhance their ability to remove pollutants. The main environmental applications and roles of surfactants discussed in this section are summarized in Table 4.

### 5.4. Quantitative Comparison of Representative Surfactant-Assisted Approaches

Quantitative comparison is a useful way to examine how surfactants affect the preparation, stabilization, and application of 2D nanomaterials. However, reported outcomes depend on several experimental factors, including the preparation method, surfactant type and concentration, purification procedure, and measurement conditions. These differences can influence the resulting nanosheet properties and make direct comparisons between studies. Therefore, this section compares representative studies using reported experimental results and considers both the benefits of surfactant-assisted processing and the limitations of comparing results obtained under different conditions. The comparison primarily focuses on graphene and GO-based systems, reflecting their versatility in surfactant-assisted processing and applications. It also includes representative energy-storage examples involving a MoS_2_/graphene hybrid and F127-modified rGO, as well as a system in which GO acts as a structure-directing surfactant for ZnO synthesis. Energy-storage applications are also represented by two additional studies in Table 5. A Gemini surfactant-assisted MoS_2_/graphene hybrid demonstrated improved lithium-ion storage performance [54], while Pluronic F127-intercalated reduced graphene oxide exhibited enhanced specific capacitance and cycling stability for supercapacitor applications [76].

The selected studies illustrate that surfactants can influence the performance of 2D nanomaterials through several mechanisms, including dispersion stabilization, surface-charge modification, interfacial interactions, and control of material morphology. For example, surfactant selection affects the electrical conductivity of graphene films after annealing [22] and the adsorption of dyes by graphene [67]. In other systems, surfactant-assisted processing enables the formation of three-dimensional GO sponges for pollutant removal [72] and GO-coated meshes with switchable wettability for oil–water separation [68]. Graphene oxide can also act as a structure-directing surfactant during ZnO synthesis, influencing its morphology, defect structure, and photocatalytic activity [35]. These examples demonstrate that surfactant effects depend on both the material system and the intended application.

Table 5 summarizes the reported processing conditions, quantitative performance metrics, and proposed mechanisms of these representative approaches. The reported values should be interpreted in the context of each study’s experimental conditions, including surfactant concentration, material composition, processing method, and performance-testing conditions. Because the studies investigate different applications and use different experimental protocols, their performance values are not directly interchangeable. Moreover, observed improvements may result from a combination of surfactant effects, changes in morphology, surface chemistry, defect structure, and interfacial interactions. Accordingly, the table emphasizes study-specific quantitative results and mechanistic interpretations rather than ranking the materials across different applications. In Table 5, NR represents “not reported” entries where the available manuscript information does not establish a value.

The comparisons in Table 5 also highlight the importance of distinguishing direct surfactant effects from changes in the structure and surface properties of the resulting materials. For example, the conductivity changes observed after thermal treatment of surfactant-stabilized graphene films may reflect both surfactant decomposition and changes in inter-sheet contacts. Similarly, the photocatalytic performance of GO–ZnO composites depends on multiple factors, including morphology, defect structure, and interfacial interactions. Comparisons with untreated materials, surfactant-treated materials, and purified materials, where reported, are therefore important for identifying the contributions of surfactant adsorption and removal. Where such controls are unavailable, the proposed mechanisms should be interpreted cautiously rather than attributed exclusively to the surfactant.

**Figure 5:**
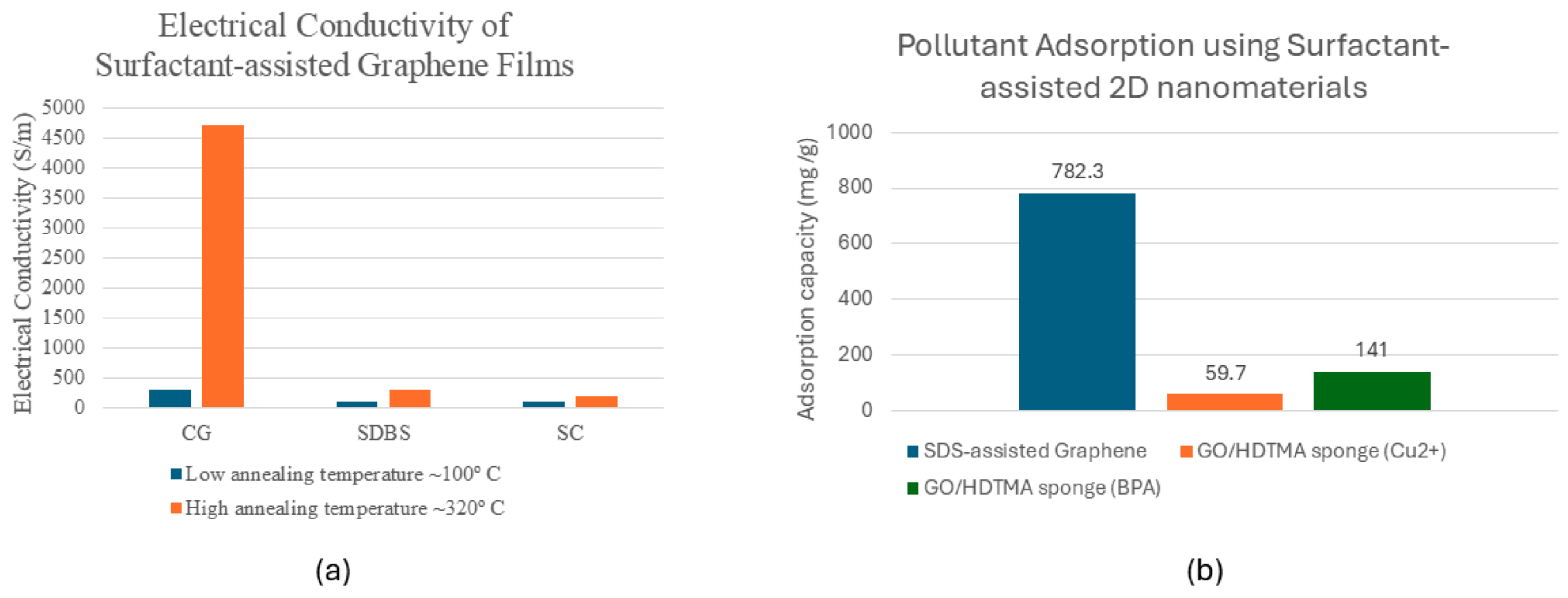
Representative performance of surfactant-assisted 2D nanomaterials. (**a**) Electrical conductivity of graphene films stabilized with coco-glucoside (CG), SDBS, and SC before and after thermal annealing. Conductivity values are approximate estimates extracted from the published graph in [22] and should not be interpreted as exact numerical values reported by the authors. (**b**) Adsorption capacities reported for SDS-assisted graphene in methylene blue adsorption and an HDTMA-modified GO sponge in Cu^2+^ and BPA adsorption. The values are taken from the respective studies cited in Table 5. Because the materials, adsorbates, and experimental conditions differ, the values are presented as representative examples rather than a direct comparison of adsorption performance. All data are based on the cited literature [67,72].

To complement the quantitative comparison in Table 5, Figure 5 presents representative performance outcomes from surfactant-assisted approaches for graphene-based materials. Figure 5a illustrates the reported changes in electrical conductivity of graphene films stabilized with different surfactants following thermal annealing, while Figure 5b compares reported adsorption capacities for selected dye and pollutant removal systems. These examples highlight the application-dependent effects of surfactant selection and modification. Because the data originate from different studies and experimental conditions, the values should be interpreted as representative performance outcomes rather than direct comparisons between materials.

## 6. Current Challenges and Future Perspectives

Although surfactant-assisted approaches have shown promise in the synthesis and engineering of 2D nanomaterials, several challenges remain. A more detailed understanding of surfactant–material interactions is needed because their effects depend on the surfactant chemistry, the type of 2D material, and the processing conditions [7,77]. For example, surfactant concentration must be carefully controlled during the liquid-phase exfoliation of transition-metal chalcogenides to promote the formation of thin nanosheets while limiting aggregation [7].

Another challenge is the removal and management of residual surfactants after synthesis or exfoliation. Depending on the material and application, these residues may alter the electrical and optical properties of the resulting materials, affect access to catalytically active sites, influence electrolyte access and ion transport, and increase the tendency of nanosheets to restack. Therefore, post-processing steps may be necessary to reduce residual surfactants [7,78]. Surfactant-stabilized graphene dispersions may require treatments such as washing, dialysis, or thermal annealing to remove surfactant molecules. However, complete removal can be difficult, and residual surfactants may remain on the graphene surface even after thermal treatment. Sethurajaperumal et al. in [22] also demonstrated that the electrical conductivity of coco-glucoside-stabilized graphene films increased substantially after thermal treatment, highlighting the potential influence of residual surfactants on electrode performance. However, the effects of post-processing should be evaluated alongside possible changes in material structure and intersheet contacts.

Recent reviews of 2D heterostructure fabrication highlight the importance of controlling residual processing agents at material interfaces. Nawaz et al. discussed the effects of transfer-polymer residues on 2D-material surfaces and summarized approaches for improving interfacial cleanliness. In a primary study, Mondal et al. used atomic force microscopy to measure residue coverage of approximately 0.08% for polypropylene carbonate (PPC) and 35.2% for polymethyl methacrylate (PMMA) on monolayer MoS_2_ [79,80]. Although these findings concern transfer polymers rather than surfactants, they highlight the importance of processing-agent residues and the need to systematically investigate how residual surfactants and their removal affect the properties and performance of 2D materials.

The required degree of surfactant removal depends on the material and its intended application. Although excess surfactant may interfere with electrical transport or block active sites, complete removal may not always be desirable if the surfactant helps maintain dispersion or structural stability. Future studies should therefore distinguish direct surfactant effects from changes associated with nanosheet thickness, morphology, defects, and interlayer spacing. Appropriate comparisons among surfactant-treated, purified, and untreated materials can help clarify these relationships.

Environmental and biological implications also require greater attention, particularly when residual surfactants may be released during material use or disposal. The toxicity and environmental impact of surfactants depend on their chemical composition and exposure conditions; therefore, these effects should not be generalized across all surfactants [24]. In addition, the environmental fate of 2D nanomaterials themselves depends on their stability, degradation pathways, degradation products, and surrounding conditions. Vranic et al. [81] emphasized the need for further investigation of the degradation and potential downstream effects of 2D materials in biological and environmental systems. Future studies should evaluate surfactant leaching, removal efficiency, recovery or reuse where feasible, and the potential for secondary contamination. Selecting surfactants that are easier to remove, biodegradable where appropriate, and less harmful to biological and environmental systems could help balance processing efficiency with long-term safety.

Scalability of 2D nanomaterial production is also a major concern. Although exfoliation and surfactant-assisted dispersion methods have shown promising results at the laboratory scale, producing large quantities of 2D materials while maintaining their quality and uniformity remains difficult. Tao et al. (2017) pointed out that while surfactant-assisted exfoliation is more scalable and environmentally friendly than many solvent-based methods, it still does not achieve production volumes suitable for industry [77]. Surfactant-stabilized dispersions are still produced at relatively low concentrations, which can limit their practical use for large-scale processing.

The reproducibility of the surfactant-assisted synthesis is also a significant factor. Factors including the concentration of the surfactant, the type of dispersant, the solvent, the processing method, and other synthesis conditions can influence the thickness, lateral size, dispersion, and quality of the nanosheets [77,82]. Even when the same materials and surfactants are used, changes in the processing conditions can cause different outcomes. It is therefore necessary to achieve more consistent control of these parameters and to gain a better understanding of their effects if the reproducibility of the surfactant-assisted synthesis is to be improved.

Future research should focus on how surfactants interact with 2D materials. Understanding these interactions can help select the best surfactants for each material and application. Recent reviews of metal-free electrocatalysts for nitrate reduction highlight the importance of surface–intermediate interactions and charge-transfer characteristics in determining catalytic performance [83]. For surfactant-assisted 2D materials, future studies should investigate how surfactant adsorption and removal affect catalytic activity and selectivity while distinguishing direct interfacial effects from changes in morphology and porosity. In practice, surfactant selection should consider the target material and application, the desired morphology and dispersion stability, the compatibility of the surfactant with the processing medium, and whether the surfactant should remain in or be removed from the final material. These criteria should be balanced against potential impacts on active-site accessibility, charge or ion transport, purification requirements, and environmental safety. Comparing these factors under clearly reported processing conditions would help establish more reliable selection guidelines across different 2D material systems. In addition, combining experiments with computational and theoretical studies can explain these processes at the molecular level and make surfactant-assisted synthesis more predictable [7]. It is also important to find better ways to control surfactant concentration and remove any excess, which could improve how well the final materials perform and how consistent they are. With more knowledge about these factors, surfactant-assisted methods could become a more reliable and scalable way to produce 2D nanomaterials with the structures and properties researchers want.

## 7. Conclusions

Two-dimensional nanomaterials offer significant opportunities in energy storage, drug delivery, environmental remediation, and other advanced applications. Surfactant-assisted engineering provides a versatile approach to controlling their synthesis, morphology, surface properties, and dispersion stability. This review examines how surfactants influence the formation and modification of 2D nanomaterials through mechanisms such as surface adsorption, template-directed growth, exfoliation, and stabilization. These interactions can affect nanosheet thickness, lateral dimensions, crystal growth, interfacial properties, and resistance to aggregation, thereby influencing the performance of the resulting materials.

The representative studies discussed in this review demonstrate that surfactant-assisted approaches can improve application-specific properties, including pollutant adsorption, oil–water separation, electrical conductivity, and photocatalytic activity. However, these improvements cannot always be attributed exclusively to surfactant action. Changes in morphology, surface chemistry, defect structure, interlayer spacing, and interfacial interactions may also contribute. Quantitative comparisons therefore need to be interpreted in the context of the experimental conditions and characterization methods used in individual studies.

The selection of a suitable surfactant should consider the target material, intended application, desired morphology and dispersion stability, compatibility with processing conditions, and whether the surfactant should remain in or be removed from the final material. These considerations must be balanced against possible effects on active-site accessibility, charge or ion transport, purification requirements, environmental safety, and production costs. Residual surfactants, inconsistent processing conditions, limited reproducibility, and difficulties in scaling up production remain important challenges.

The comparison across processing strategies and applications indicates that surfactant retention or removal should be determined by the intended function of the surfactant and the requirements of the final material. Retaining adsorbed surfactants may be beneficial when continued dispersion stability or surface modification is required, whereas reducing or removing residual surfactants may be preferable when they obstruct active sites, impede charge or ion transport, or increase electrical resistance. For structure-directing approaches, surfactants may be removed after synthesis when the desired architecture can be preserved. These choices are not universal: the effects depend on surfactant chemistry, the 2D material, processing conditions, and the target application. Moreover, the available studies do not always isolate surfactant-specific effects from simultaneous changes in morphology, defects, interfacial structure, or electrode contacts. Controlled comparisons that characterize residual surfactants and evaluate performance before and after purification are therefore needed to establish more reliable selection criteria, because the available studies do not consistently isolate the effects of surfactant removal from concurrent changes in material structure and interfacial contacts.

Future research should establish more systematic relationships between surfactant chemistry, processing conditions, material structure, and application performance. Greater attention is also needed to surfactant recovery and removal, environmental fate, long-term material stability, and reproducible performance under practical operating conditions. Combining controlled experiments with computational and theoretical studies may help clarify surfactant–material interactions and guide more effective surfactant selection. Overall, advancing surfactant-assisted engineering will require not only improved material performance but also reliable processing, appropriate environmental assessment, and scalable production strategies.

## Figures and Tables

**Table 1:** Common types, key properties, applications, and relevance of two-dimensional nanomaterials to surfactant-assisted synthesis.

Property	Graphene	h-BN	TMDs	MXenes
Structure	Single-layer carbon atoms in a hexagonal honeycomb structure	Hexagonal lattice of alternating B and N atoms	Transition-metal layer sandwiched between chalcogen layers	Layered structure with tunable surface terminations
Key properties	High electrical conductivity, strength, large surface area	High thermal stability, electrical insulation, chemical stability	Tunable electronic/optical properties, high flexibility	High electrical conductivity, large surface area, tunable surface chemistry
Major applications	Energy storage, sensors, electronics	Dielectrics, lubrication, electronics	Sensors, energy storage, optoelectronics	Energy storage, sensors, catalysis, water treatment
Relevance to surfactants	Dispersion, exfoliation, surface modification	Exfoliation and dispersion	Morphology and growth control	Surface modification, dispersion, adsorption

**Table 2:** Major Mechanisms and functions of Surfactant-Assisted Synthesis and Morphology Control of 2D Nanomaterials.

Mechanism	Representative Surfactants/Additives (Classified by Type)	Role in Synthesis or Processing	References
Template-Directed Synthesis	Conventional surfactants: SDS, SOS, CTAB	Provides a soft or solid template for precursor organization and directs the formation of ultrathin structures	[31,32,33,34]
Interface-Assisted Synthesis	Conventional surfactants: SDS and other anionic surfactant monolayers and bilayers	Creates an organized reaction environment and order precursor accumulation at charged interface	[9,26]
Crystal Growth Regulation	Conventional surfactants: CTAB, SDS; Polymeric additive/stabilizer: PVP	Influence growth kinetics, facet development, and crystal growth; PVP can restrict vertical stacking and stabilize nanosheets.	[6,27,28]
Morphology Control	Conventional surfactants: SDS, CTAB, CTAC; Polymeric additives: PVP, PEG; Coordinating agent: EDTA; 2D macromolecular surfactant: GO	Control thickness, lateral dimensions, shape, aspect ratio, and structural uniformity through different mechanisms, including growth regulation, coordination, and structure direction.	[7,28,31,33,35,36]
Liquid-Phase Exfoliation	Conventional surfactants: SDS, SDBS	Promotes dispersion and stabilization and reduces reaggregation	[29,37,38]
Dispersion and Stabilization	Conventional surfactant: SDBS; Polymeric stabilizer: PVP; 2D macromolecular surfactant: GO	Reduce nanosheet aggregation or restacking and help maintain stable dispersions through system-dependent stabilization mechanisms.	[28,29,35,38,39]

**Table 3:** Summary of surfactant-engineered 2D nanomaterials for drug delivery.

2D Nanomaterial	Surfactant/Surface Engineering	Role in Drug Delivery	References
Graphene/GO	Surfactant stabilization; FA functionalization (targeting ligand, not surfactant)	Improves dispersion and drug loading; enables targeted and controlled drug delivery	[23,41,43,49]
MoS_2_/TMDs	Surfactant-assisted exfoliation and stabilization	Prevents restacking and provides accessible surfaces for drug loading and triggered release	[23,30,40,50]
Black phosphorus (BP)	HSA-assisted exfoliation and capping	Improves nanosheet preparation and facilitates PTX loading through hydrophobic interactions	[23,40,42]
2D MOFs nanosheets	PVP-assisted synthesis	Controls nanosheet structure and provides accessible sites for drug loading and release	[51]
Porphyrinic 2D MOFs	Structural/surface engineering	Enables loading and controlled delivery of drugs and photosensitizers	[48,51]
Stimuli-responsive 2D nanocarriers	Structural/surface engineering	Enables pH-, redox-, or externally triggered drug release	[40,49]
MXenes	PEG surface modification	Improves colloidal stability and physiological compatibility	[44,45]

**Table 4:** Surfactant-assisted 2D nanomaterials for environmental remediation.

Environmental Application	Representative Surfactants/Additives	Role of Surfactants/Additives	References
Photocatalytic degradation	Synthesis additives: Urea, oxalic acid; Polymeric additive: PVP; Conventional surfactants: CTAB, detergent-derived surfactants	Improves exfoliation, dispersion, morphology, and surface properties, contributing to photocatalytic activity	[65,66,70]
Organic pollutant adsorption	SDS, CTAB, Pluronic F108; CTAB modification	Modifies surface charge, dispersion, and interlayer spacing, influencing pollutant–surface interactions	[67,71]
Simultaneous pollutant removal	HDTMA-Br	Modifies GO surface interactions and promotes formation of a stable 3D sponge structure	[72]
Oil/water separation	CTAB, SDS, Polymeric additive: PVP	Modifies wettability or interfacial interactions to enable selective oil/water separation	[68,73]

**Table 5:** Quantitative comparison of representative surfactant-assisted engineering of 2D nanomaterials.

2D Material and Surfactant	Quantitative Structural and Processing Data	Quantitative Performance	Interpretation and Limitations	References
Graphene stabilized by coco-glucoside (CG), SC, and SDBS	Graphite: 10 g; surfactant: 400 mg; water: 100 mL; probe sonication: 1 h at 60% amplitude; centrifugation: 500 rpm for 90 min (concentrated-dispersion preparation). CG-stabilized graphene: average lateral dimension approximately 1 μm; average layer number 6 ± 4. CG zeta potential: approximately −22.3 mV. Quantitative yield: NR.	CG-stabilized graphene film: 15-fold conductivity enhancement after annealing at 320–400 °C compared with films annealed at 100–200 °C. Approximately 90% of CG decomposed at 320 °C. SC- and SDBS-stabilized films showed only marginal conductivity changes under the reported conditions.	Surfactant decomposition may improve electrical contacts between graphene nanosheets. However, changes in film morphology and nanosheet connectivity may also contribute. SC and SDBS provide comparative controls; a surfactant-free film control is NR in the available information.	[22]
Graphene—SDS, CTAB, Pluronic F108	Graphite suspension: 2% *w*/*w*; SDS solution: 0.1% *w*/*w*, added gradually over approximately 48 h under ultrasonication. Graphene suspensions were centrifuged at 2500 rpm for 20 min and dialyzed for at least 48 h to remove unadsorbed surfactant. Nanosheet dimensions, yield, and quantitative dispersion stability: NR.	SDS-exfoliated graphene achieved a maximum methylene-blue adsorption capacity of 782.3 mg/g at 25 °C. Adsorption approached equilibrium within approximately 10 min under the tested conditions. Isotherm tests: pH 7, 60 rpm, initial dye concentration 5–150 mg/L. Adsorbent dose and precise mass basis of the reported capacity: NR in the available information.	Adsorption performance depends on surfactant identity and dye charge. SDS gives graphene a negatively charged surface that favors adsorption of positively charged methylene blue through electrostatic attraction. The reported capacity applies specifically to methylene blue under the study’s experimental conditions.	[67]
GO sponge modified with HDTMA-Br	HDTMA content: 6.4–11.5% associated with hydrogel formation and stable sponge formation. The tested GO/HDTMA sponge contained 10.5 wt% HDTMA; prepared by stirring at 400 rpm, ultrasonication for 1 h, rinsing twice with deionized water, and freeze-drying. Nanosheet dimensions and synthesis yield: NR.	Maximum adsorption capacities: 59.7 mg/g for Cu^2+^ and 141.0 mg/g for BPA. Approximately 100% removal was reported within 1 h for Cu^2+^ and 2 h for BPA. Isotherm tests: pH 5, 25 °C, 180 rpm, initial concentrations of 0–100 mg/L; kinetic tests used 20 mg/L of each pollutant. Simultaneous-adsorption dose: 10 mg in 30 mL of solution. The capacities are reported for the GO/HDTMA composite adsorbent; the precise mass basis is not explicitly specified in the original study.	HDTMA promotes formation of a stable 3D GO sponge and substantially improves BPA adsorption through hydrophobic interactions. However, it may reduce Cu^2+^ adsorption by occupying negatively charged adsorption sites. Unmodified GO and GO/chitosan serve as comparative controls	[72]
GO-coated metal mesh—CTAB	GO: 0.1 g dispersed in 50 mL deionized water by sonication for 2 h. CTAB solutions: 0.5, 1, 2, and 4 g/L. GO–CTAB complex electrodeposition: 0.2 A for 20 min. The GO and CTAB concentrations of 1 g/L each provided the best reported switchable-wettability performance. Mesh dimensions, coating mass, and number of mesh samples: NR.	Separation flux exceeded 450 L m^−2^ h^−1^ for oil-in-water emulsions and was approximately 1800 L m^−2^ h^−1^ for a water-in-oil emulsion. Wettability switching was repeated for at least 10 cycles without significant deterioration. Immiscible seawater–light-oil mixtures were separated with flux above 130,000 L m^−2^ h^−1^ under the reported conditions. Separation efficiency (%): NR in the available information.	CTAB–GO interactions and reorientation of CTAB chains change surface wettability, enabling reversible separation. The unmodified GO-coated mesh did not exhibit the same wettability-switching ability and serves as a control.	[68]
GO as a 2D macromolecular surfactant for ZnO	GO loading: 1, 3, 5, and 10 wt%. Synthesis in aqueous solution at 90 °C for 3 h. The 3 wt% RGO–ZnO composite formed a hollow, hexagonal tube-like structure with a wall thickness of approximately 20 nm and diameter of approximately 2 μm. GO nanosheet dimensions and product yield: NR	Photocatalytic Cr(VI) reduction was evaluated using 30 mg catalyst in 60 mL of 5 ppm Cr(VI) solution under visible-light irradiation (λ > 400 nm). The 3 wt% RGO–ZnO composite exhibited the highest apparent rate constant, approximately 0.17 h^−1^, compared with approximately 0.10, 0.15, and 0.085 h^−1^ for the 1, 5, and 10 wt% composites, respectively. Bare ZnO showed negligible visible-light activity under the reported conditions.	GO influences ZnO morphology and defect structure and promotes oxygen-vacancy formation. The blank ZnO and GO-free graphene–ZnO control help distinguish the roles of GO functional groups and oxygen vacancies. The improved activity cannot be attributed to surfactant action alone.	[35]
MoS_2_/graphene hybrid; Gemini 12-3-12 surfactant	Gemini surfactant: 0.8 mmol; graphene oxide suspension: 30 mL (3.0 mmol); hydrothermal treatment: 240 °C for 24 h; annealing: 800 °C for 2 h under 10% H_2_/N_2_. MoS_2_ nanosheets: 3–6 layers; MoS_2_ content: 65.38 wt%. Lateral dimensions, yield, and long-term dispersion stability: NR.	At 100 mA g^−1^: initial reversible capacity of 1127 mAh g^−1^; 1096 mAh g^−1^ after 100 cycles (97.5% retention) and 1033 mAh g^−1^ after 220 cycles (91.6% retention). At 1000 mA g^−1^: approximately 760–770 mAh g^−1^.	Promising for lithium-ion battery energy storage, with improved capacity retention and rate capability versus pristine MoS_2_. Performance reflects combined effects of graphene incorporation, hybrid morphology, and surfactant-assisted synthesis; the surfactant’s independent contribution was not isolated.	[54]
Reduced graphene oxide (rGO); Pluronic F127 triblock copolymer	F127: 12.5 mg; water: 20 mL; GO solution: 5 mL (5 g L^−1^); stirred for 24 h before and after GO addition; hydrothermal treatment at 180 °C for 12 h; annealing at 400 °C under Ar. BET surface area: 696 m^2^ g^−1^; pore volume: 2.34 cm^3^ g^−1^. Nanosheet dimensions, quantitative yield, and long-term dispersion stability: NR.	Specific capacitance: 210 F g^−1^ at 1 mV s^−1^ and 168 F g^−1^ at 1 A g^−1^ in 6 M KOH. Capacitance retention: 95.6% after 1000 cycles at 1 A g^−1^. Pristine rGO: 101 F g^−1^ at 1 A g^−1^.	Demonstrates potential for supercapacitor energy storage, with enhanced capacitance and cycling stability versus pristine rGO. Improvements are associated with increased surface area, pore volume, and wettability; excessive annealing can reduce performance as F127 decomposes.	[76]

## Data Availability

No new data were created or analyzed in this study. Data sharing is not applicable to this article.

## Abbreviations

The following abbreviations are used in this manuscript:

2D	Two-dimensional
AE	Alcohol ethoxylates
AO	Amine oxide
APE	Alkylphenol ethoxylate
BAC	Benzalkonium chloride
BP	Black phosphorus
BPA	Bisphenol A
CAGR	Compound Annual Growth Rate
CG	Coco-glucoside
CMC	Critical Micelle Concentration
CNT	Carbon nanotube
CTAB	Cetyltrimethylammonium bromide
CTAC	Cetyltrimethylammonium chloride
CVD	Chemical vapor deposition
DMSO	Dimethyl sulfoxide
EB	Egyptian blue
EDTA	Ethylenediaminetetraacetic acid
GO	Graphene oxide
h-BN	Hexagonal boron nitride
HDTMA	Hexadecyltrimethylammonium
HDTMA-Br	Hexadecyltrimethylammonium bromide
HSA	Human serum albumin
LAS	Linear alkylbenzene sulfonate
LDHs	Layered double hydroxides
LIB	Lithium-ion battery
MB	Methylene Blue
MOFs	Metal–organic frameworks
MR	Methyl Red
PEG	Polyethylene glycol
PPC	Polypropylene carbonate
PMMA	Polymethyl methacrylate
PVP	Polyvinylpyrrolidone
QAC	Quaternary ammonium compound
rGO	Reduced graphene oxide
SC	Sodium cholate
SDS	Sodium dodecyl sulfate
SDBS	Sodium dodecylbenzene sulfonate
SIB	Sodium-ion battery
SOS	Sodium octadecyl sulfate
TMDs	Transition metal dichalcogenides
TMO	Transition-metal oxide
